# An evolutionary perspective on social inequality and health disparities: Insights from the producer–scrounger game

**DOI:** 10.1093/emph/eoad026

**Published:** 2023-08-16

**Authors:** Jonathan C K Wells

**Affiliations:** Childhood Nutrition Research Centre, Population, Policy and Practice Research and Teaching Department, UCL Great Ormond Street Institute of Child Health, London, UK

**Keywords:** social inequality, nutrition, racism, gender inequality, life history theory, game theory

## Abstract

There is growing concern with social disparities in health, whether relating to gender, ethnicity, caste, socio-economic position or other axes of inequality. Despite addressing inequality, evolutionary biologists have had surprisingly little to say on why human societies are prone to demonstrating exploitation. This article builds on a recent book, ‘*The Metabolic Ghetto*’, describing an overarching evolutionary framework for studying all forms of social inequality involving exploitation. The dynamic ‘producer-scrounger’ game, developed to model social foraging, assumes that some members of a social group produce food, and that others scrounge from them. An evolutionary stable strategy emerges when neither producers nor scroungers can increase their Darwinian fitness by changing strategy. This approach puts food systems central to all forms of human inequality, and provides a valuable lens through which to consider different forms of gender inequality, socio-economic inequality and racial/caste discrimination. Individuals that routinely adopt producer or scrounger tactics may develop divergent phenotypes. This approach can be linked with life history theory to understand how social dynamics drive health disparities. The framework differs from previous evolutionary perspectives on inequality, by focussing on the exploitation of foraging effort rather than inequality in ecological resources themselves. Health inequalities emerge where scroungers acquire different forms of power over producers, driving increasing exploitation. In racialized societies, symbolic categorization is used to systematically assign some individuals to low-rank producer roles, embedding exploitation in society. Efforts to reduce health inequalities must address the whole of society, altering producer–scrounger dynamics rather than simply targeting resources at exploited groups.

## INTRODUCTION

There is escalating concern over social disparities in health, whether these relate to gender, ethnicity, indigeneity, migration, caste, socio-economic position, education or other axes of inequality [1, 2]. These concerns fit squarely within the discipline of public health, which aims to promote health and reduce disease through the organized efforts of society [3]. However, the issue of social inequality goes far beyond the arena of health, as inequalities also pervade social experience, economic livelihood and self-expression, ultimately relating to fundamental concepts of freedom and human rights. Groups routinely subjected to different forms of social subordination and discrimination pay multiple penalties.

For most sources of ill-health, scientists aim to dissect the underlying aetiology dispassionately, so that resources for prevention and treatment can be targeted effectively. Any strategy that reduces disease is likely to receive widespread support. In contrast, concerns with health disparities take on a unique moral dimension, as they are structurally embedded in the functioning of societies that systematically distribute risks, costs and benefits unequally. Conceptual frameworks that address societal inequality are generally considered the domain of political rather than natural sciences, and a moral dimension is explicitly brought to the forefront.

Political sciences aim to mobilize opinion, and incorporate ideology in order to challenge the status quo and overcome resistance to change. Common to movements such as feminism and Black Lives Matter, for example, is the idea that to overcome vested interests, the whole of society must change. In contrast, public health approaches to health disparities often pay little attention to healthier dominant groups, and instead aim to address the unequal distribution of disease by targeting interventions at high-risk groups, without changing society itself. Examples include cash-transfer programmes for those with low income [4–6], or focussing public health campaigns in deprived neighbourhoods [7–10].

Consequently, despite the high prevalence of societal disparities in health [11–13], there are few scientific frameworks for understanding *why* human societies are generically hierarchical, and *why* the same groups in diverse societies routinely experience disadvantage. In turn, this hinders the development of novel and effective strategies to reduce health disparities. Moreover, those most adversely affected have often been presented by intellectuals and governments as ‘problem groups’ that are responsible for their own misfortune. In the 19th century, for example, poorer groups were considered a drain on the ‘more productive’ members of society [14], and the notion of the poor as ‘scroungers’ remains pervasive in modern economic policies [15]. Similar representations have been provided for other groups that experience persistent subordination, including women and racialized groups [16, 17].

The aim of this review is to present an opposing perspective, highlighting the exploitation of subordinated groups. Drawing on evolutionary dynamic game theory, I provide a unifying scientific framework for understanding how social inequalities emerge and persist in societies, maintained by power relations. This approach focuses on how those with more resources systematically gain them through exploiting those who produce them. I update a perspective previously outlined in my book ‘*The Metabolic Ghetto*’ [18], to present it to a wider audience. As the supporting evidence has been reviewed in detail [18], my aim here is to present the overarching theoretical framing, to show how it can be widely applied.

Hierarchical groups are not exclusive to humans, and can be seen in animals spanning social insects to chimpanzees [19–23]. This makes social hierarchy a prime topic for an evolutionary approach, and yet relatively few researchers have worked in this area. The evolution of human cooperation has attracted substantially more attention from anthropologists [24–27] than relations of inequality and conflict, perhaps because hunter-gatherer societies tend to show relatively low levels of hierarchy, conflict and inequality [28–30]. The primary evolutionary perspective on social inequality and hierarchy has come from the subdiscipline of Human Behavioural Ecology. As **Box 1** highlights, this approach has primarily focussed on inequalities in access to material resources (such as land, technology and food) and their consequences, and has paid less attention to the direct exploitation of people. The approach described here aims to address this gap.

**Box 1. Evolutionary approaches to social inequality, hierarchy and exploitation**

The main evolutionary perspective on human inequality and hierarchy has emerged through the sub-discipline of Human Behavioural Ecology (HBE) [28, 137, 144]. This approach links behavioural variation with environmental variability, on the assumption that individuals will adjust their behaviour to the environment through reaction norms in ways that maximize fitness. It is the reaction norm, not the behaviour itself, that is subject to selection [144].

HBE was initially used to address the emergence of social groups, whereby individuals seek to maximize fitness by accessing limiting resources through relationships of cooperation or competition [145]. Subsequent applications explored how social inequalities and hierarchies could emerge in such groups. Boone considered how in hierarchical societies, dominants compete with other dominants to optimize the number of subordinates over which they have control, while the subordinates compete with each other to maximize their access to the resources controlled by the dominants [145]. Kelly reviewed evidence for slavery being used to promote household production in past sedentary forager populations from the North West American coast [28].

Key issues in the HBE approach to hierarchy are (i) the defensibility of resources, whereby productive resources favour individuals trying to prioritize their access, and (ii) the relative bargaining power of dominant and subordinate groups, which shapes the competition over the resources [137, 144]. Reproductive skew is another critical variable, as in certain conditions males may seek to maximize their control of resources to benefit the fitness of themselves and their kin, resulting in despotism [146]. Ecological conditions may shape the potential for despotism through differential impacts on the productivity and frequency of males and females in the population [146, 147]. There is a long tradition of applying dynamic game theory to behavioural ecological issues [148].

While overt relationships of exploitation such as slavery are acknowledged in such literature [28, 147], the primary emphasis in HBE is on competition for physical resources (land, technologies of production, food items) that are separate from people themselves. The approach highlighted in this article, based on the producer–scrounger game [40], differs in that its focus is directly on human labour, and hence the exploitation of producers’ foraging effort by scroungers. Through this lens, hierarchical societies emerge when scrounging becomes viable, and they consolidate when dominants increasingly force producers to perform diverse forms of work for them. Beyond differential access to resources *per se*, some of the resources extracted from producers are converted by scroungers into physical factors and social institutions that maintain the overall system of exploitation [18]. Since scroungers by definition are unproductive, the maintenance of exploitative institutions is paradoxically funded entirely by the exploited. This approach complements other HBE approaches to hierarchy, and continues the tradition of drawing on dynamic game theory.

In hierarchical societies, things have gone badly for some, but relatively well for dominant individuals who have a vested interest in maintaining the status quo. It has been noted that highly stratified societies impose some costs on all of their members [31, 32], but the vast majority of costs and benefits are unequally distributed. Instead of trying to solve the problems of subordinate groups in piecemeal manner without challenging the privileges enjoyed by dominant groups, I argue that we need to consider how societal dynamics need to change. In this approach, both dominant and non-dominant groups should be the target of interventions, with the aim of changing how these groups interact.

This approach relates to others that apply social niche construction [33, 34] as a lens for understanding various aspects of inequality [35–38]. Human niche construction involves not only physical effects on the environment, but also the construction of long-lasting social institutions that shape the socio-ecological environment into which individuals are born and live their lives [35]. Niche construction theory has been used to examine how changes to the current niche (e.g. social institutions) may impact the fitness of subsequent generations [39]. However, few studies have so far attempted to probe *how* human hierarchy and exploitation develop through niche construction.

Human societies are always ultimately ‘food systems’, and as described below, my approach centres all forms of exploitation within the different types of power relations that characterize social food systems [18]. Even in animals, dominant individuals may control subordinates using several strategies, including physicality, threat or violence, behavioural manipulation or social alliances. For humans, however, ideology is an additional mechanism, and arguably the most important. Without the large human brain, capable of symbolic representation, societies would be incapable of developing and perpetuating the structured and exploitative forms of hierarchy discussed below.

## THE PRODUCER–SCROUNGER GAME

The key insight of game theory for societal dynamics is that the best behavioural strategy for any individual is not fixed, but depends on what others in the population are doing. Both the costs and pay-offs of any strategy are dependent on the actions of others, hence ‘decisions’ must constantly be updated, and tailored to prevailing social and ecological conditions. Various dynamic games have been developed for evolutionary application, such as *hawk-dove*, the *ultimatum game* and the *prisoners’ dilemma*, but I focus here on the ‘*producer-scrounger*’ (P–S) game, developed by ecologists to understand social foraging [40, 41].

The basic premise of the P–S game is that each individual may either produce resources, or steal or ‘scrounge’ them from others that have already produced them. The game assumes a social group, within which each individual, at any given time, may pursue only one of the strategies [42]. Across time, however, individuals may alter their strategy depending on the nature of the pay-offs.

**Figure 1.**
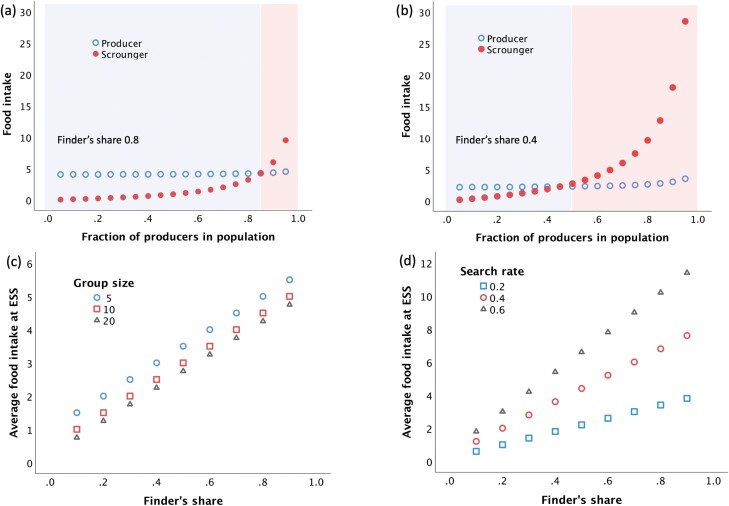
Basic producer–scrounger game, using the equations of Barta [43]. (a) Food intake for producers and scroungers, for different proportions of producer in the population, assuming fixed group size and searching rate and a high finder’s share of 0.8. The evolutionary stable strategy (ESS) occurs when 85% of individuals produce. Below this frequency of producers, producers have higher returns than scroungers (blue shaded area), whereas above it the opposite holds (red-shaded area). (b) Food intake in the same population, with a lower finder’s share of 0.4. Now the ESS occurs when only 45% of the group produce, as the pay-off is lower. (c) Effect of group size on the average food intake of producers and scroungers at the ESS, in relation to the magnitude of the finder’s share. (d) Effect of searching rate on the average food intake of producers and scroungers at the ESS, in relation to the magnitude of the finder’s share. The equations are given in the Supplementary Appendix.

Simple representations of the game highlight several key points. First, an evolutionary stable strategy (ESS) is predicted to emerge, where there is a stable equilibrium between producers and scroungers in terms of pay-offs. When producers are common and scroungers rare, any individual producer could potentially benefit by switching strategy. As more producers switch to scrounging, however, the overall productivity of the group falls, as do the returns for individual scroungers. Above a certain threshold of scroungers, it thus becomes better to revert to producing (**Fig. 1a**) [40]. The ESS identifies the point where neither producers nor scroungers would benefit from switching strategy. In practice, an ESS might involve either certain proportions of individuals always producing or always scrounging, or every individual using both strategies for certain proportions of the time [44]. For the parameters illustrated in Fig. 1a, the ESS occurs when the fraction of producers is 0.85.

Scroungers outsource the costs of foraging to producers, but this strategy will only succeed if there is anything to scrounge [45]. A major incentive for producing food is known as the ‘finder’s share’, a portion that can be consumed quickly before any scroungers arrive [43]. In Fig. 1a, the finder’s share comprised a high proportion (0.8) of the food. As the finder’s share falls, producing becomes less rewarding, and the threshold for switching to scrounging declines. When the finder’s share is 0.4, for example, the ESS occurs when the fraction of producers is only 0.45 (**Fig. 1b**). While scrounging is now more rewarding, however, the intake for each strategy at the ESS is lower, because less food is produced overall. The rewards for producing thus affect both parties’ intakes.

Returns also depend on group size. Each producer finds food at a certain rate, whereas scroungers contribute nothing. As group size increases, the number of individuals seeking a ‘piece of pie’ increases at a steeper rate than the total ‘volume of pie’ produced. Larger groups therefore result in lower average food intake for both strategies at the ESS (**Fig. 1c**). If the rate of producing increases, in contrast, then so does the average intake of both parties, and this occurs disproportionately when the finder’s share is greater because this motivates more individuals to produce (**Fig. 1d**).

Applications of the game to ecological data strongly support it [42, 44, 46], and highlight several factors that shape P–S interactions and the choice of strategy by individuals. Much evidence derives from studies of birds, as many species obtain discrete food packages that can easily be stolen, but studies of mammals also provide support [47, 48].

First, when faced by scroungers, producers tend to offer only limited effort to defend their resource. Possession of a food package may impede the producer’s mobility, hence the costs of defence might include injury, while the chances of retaining the food may be low. This then reduces the potential costs of scrounging, especially for agile individuals [42]. Second, species that scrounge from other species may be larger in size, or more aggressive, but another notable feature is that they often have relatively large brains, suggesting that they use cunning in deciding how to scrounge [42]. Finally, chasing producers is only worthwhile if there is a good chance of obtaining food. Scrounging is favoured when food items are discrete, visible, nutritious and took effort to find [45]. There is no benefit to scrounging a ubiquitous resource, such as grass on a savannah.

Applying the P–S game to humans brings the food system into central focus, though other resources are also relevant including access to mates or status goods. We can now ask, using this simple dynamic model, how P–S dynamics emerge and stabilize in larger societies.

## DIVERGENT STRATEGIES

Early P–S models assumed that any animal might select a strategy on an *ad hoc* basis, depending on the immediate rewards and what others were doing. However, certain phenotypic characteristics might systematically favour one strategy, so that individuals might become a routine producer or scrounger [49].

Initially, such variation might relate to behaviour, for example animals with more aggressive or cunning personalities might systematically favour scrounging. In studies of Mexican Jays, dominant birds tended to join up with subordinate birds, who were thus routinely prone to being taxed of their foraging effort [50]. Studies of animal personalities have shown that traits relevant to scrounging (exploration, boldness, aggression) tend to cluster within individuals, as they are all related to risk-taking [51].

Such behavioural contrasts might then impact physical traits. If aggressive scroungers routinely allocate part of their energy dividend to growth, for example, they can develop larger body size [52]. In many species, size determines the outcome of social competition, as animals generally prefer to threaten aggression rather than enact it, to avoid injury. Larger scroungers carry a reliable advantage in interactions with producers, including in direct competition over food as modelled in the *hawk-dove* game [53].

The benefits of large size in gaining access to resources are evident in inter-species ‘pecking orders’, but the same dynamics relate to members of a social group. In a study of coho salmon, for example, smaller fish were typically producers while large fish showed higher frequencies of scrounging, particularly when groups incorporated wide variability in body size [54]. Other components of phenotype that are stable at any time point include age and gender, hence we may likewise expect these traits to shape P–S tactics. In female primates, for example, rank is often associated with the number of social alliances [55, 56] rather than body size, with implications for scrounging tactics [47].

The P–S game assumes that at equilibrium, the pay-off of both parties is equal. Over time, their Darwinian fitness must also match, otherwise a new ESS would emerge. However, this does not mean that all characteristics (including food intake) are also identical. Rather, when individuals systematically adopt one strategy, differences in phenotype and experience may consolidate. The impact of this scenario on health outcomes can be understood using life history theory.

Life history theory assumes that all organisms are under selective pressure to allocate their energy in competition between four functions, termed *maintenance*, *growth*, *reproduction* and *defence* [57, 58]. The more energy allocated to one function, the less available for others, resulting in allocation ‘trade-offs’.

Individuals that routinely produce or scrounge may make contrasting trade-offs, driving divergent life history profiles [18, 59]. Watve, for example, modelled humans as two groups with high or low aggression, generating contrasting metabolic phenotypes [59]. While P–S strategies may always be selected under the pressure to maximize fitness, the underlying variability in growth, longevity and fertility relates directly to health outcomes.

As reviewed in ref. [18], life history contrasts between individuals of high and low social rank emerge in particular during three life-course periods, demonstrating systematic penalties for those of lower rank:

- poor survival and growth in early life [60–64]
- higher fertility, to compensate for greater offspring mortality [65, 66]
- faster ageing and shorter lifespan [67, 68]

Such associations are often also evident in other social species. Among primates, for example, studies show that higher-ranked mothers have priority access to better foraging patches, mature faster, and produce larger offspring with higher survival rates compared to their low-ranked peers [69]. However, which of dominant and subordinate individuals show faster ageing in adult life may depend on the species and the social system [22]. In baboons, for example, high status males were shown to have faster epigenetic ageing [70], whereas in human populations, epigenetic age tends to be greater among those of low social status [67, 68]. One reason why those of lower rank may experience the greatest penalties in humans is that producers can rarely vote with their feet and opt out of exploitative situations.

Though the structures of human societies are different, similar patterns are evident. To link with the P–S model, we can identify discrete groups in society that map onto scroungers and producers. The neighbourhood ‘deprivation index’ scales closely with both household income and public resources, hence the top and bottom deciles of this index may be considered to identify the two groups. In contemporary England, those living in the most deprived neighbourhoods have lower birth weight, shorter gestational age, greater early mortality, shorter childhood height and lower healthy and total lifespans, compared to those living in the least deprived neighbourhoods (**Table 1**) [71–73]. However, Table 1 also shows a social gradient for all outcomes, suggesting a need for more complex models of society as a social ladder, where individuals are exploited by those above them, and exploit those below them.

**Table 1.** Life history and demographic outcomes in the English population, according to the level of neighbourhood deprivation

Health outcome	Decile of multiple deprivation index (1 = poorest)									
	1	2	3	4	5	6	7	8	9	10
Low birth weight (%)	10.5	-	-	-	-	-	-	-	-	3.9
Preterm birth per 1000	6.7	6.3	6.0	5.7	5.3	5.2	5.0	4.9	4.8	4.5
Neonatal mortality per 1000	2.8	2.6	2.5	2.1	1.9	1.8	1.6	1.6	1.5	1.5
Infant mortality per 1000	4.7	4.1	3.8	3.3	2.9	2.7	2.4	2.3	2.2	2.1
Short stature in childhood (%)	2.6	2.2	2.1	2.0	1.9	1.7	1.7	1.6	1.5	1.4
Healthy life expectancy^a^ (M)	52.3	56.4	58.4	61.1	64.1	65.1	65.9	67.3	68.5	70.5
Healthy life expectancy^a^ (F)	51.9	56.8	59.6	62.0	64.2	65.1	67.3	68.5	69.5	70.7
Life expectancy^a^ (M)	73.5	75.8	77.3	78.6	79.7	80.4	81.0	81.6	82.1	83.2
Life expectancy^a^ (F)	78.3	80.2	81.5	82.4	83.3	83.9	84.4	84.8	85.3	86.3

The table emphasizes two extreme groups (grey-shaded) in the multiple deprivation index, which can be considered conceptually to represent the discrete groups termed producers (decile 1) and scroungers (decile 10) that are modelled in the dynamic producer–scrounger game. However, where further data are available, the table also illustrates relatively linear social gradients in all outcomes between the highest and lowest deciles. Based on data from Opondo *et al*. [71], Orr *et al*. [72] and UK Census 2021 [73].

^a^Life expectancy at birth.

Across generations, phenotype may reproduce itself, mediated by contrasting levels of maternal investment and the socio-economic niche. In a Brazilian birth cohort, a composite index of maternal capital (reflecting adult height, body mass index, education, household income) showed dose–response associations with life history traits of the offspring, including early growth, adult height and body composition, schooling and the timing of reproduction. Both sons and daughters of low-capital mothers tended to replicate their mothers’ adverse health and social outcomes [74, 75].

**Figure 2.**
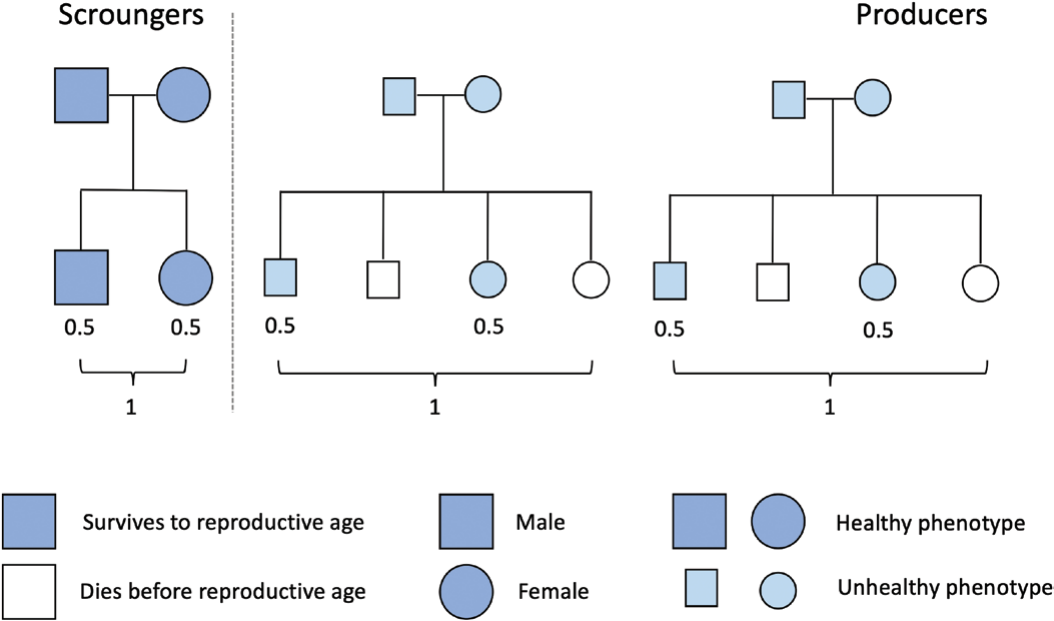
Schematic diagram illustrating fitness and demographic outcomes in a hypothetical population of producers (common and subordinate) and scroungers (rare and dominant). Scroungers have negligible juvenile mortality and produce small numbers of high-quality offspring. Producers have high juvenile mortality, and compensate through higher fertility rates. The total fitness of producers and scroungers is similar, but producers have poorer health and shorter average lifespan. Adapted with permission from Wells, 2023 [76].

The intergenerational transmission of social rank leads to ‘dynasties’ of producers and scroungers, the basis of stratified societies. In a hypothetical stable population, health differences are predicted to emerge even when average producers and scroungers achieve equal Darwinian fitness, namely replacing themselves (**Fig. 2**). Scroungers may achieve this via low juvenile mortality, low fertility, large size and high longevity, and producers via contrasting characteristics, representing intergenerational cycles of advantage and disadvantage, respectively. In this way, the P–S game collectively underpins the transmission of the entire social system, social rank and health inequalities across generations [18, 76].

The health outcomes in Table 1 all have associations with nutrition. Size at birth and gestational age indicate maternal investment *in utero*, while childhood height reflects post-natal nutrition among other factors. Healthy and total lifespans reflect cardiometabolic health, in turn related to early growth patterns and adult adiposity. A key point in my argument is that, precisely *because* the P–S game plays out in the production and acquisition of food and nutrients, the health outcomes affected by hierarchy relate strongly to nutritional status through the life course [18].

As highlighted in **Box 1**, the P–S game is essentially a model of the exploitation of effort. When scroungers acquire resources, they gain them as the fruits of others’ labour. If we think of the total volume of resources produced as ‘the pie’, however, the scroungers not only end up with more pie per capita, but also invest some of their extra resources in maintaining the system of exploitation. I now focus on three forms of inequality involving exploitation where the P–S game provides new integrative insight. This is not an exhaustive list, my aim is simply to show how a single theoretical approach can be widely applied.

## GENDER DYNAMICS

Gender inequality is particularly troubling, because its ubiquity in different forms across societies might seem to indicate that it is in some way ‘natural’, a legacy of human evolution. For this reason, the late 20th century saw a shift away from biological enquiry into gender inequality, to avoid posing any form of justification [77]. The feminist movement instead prioritizes active efforts to confront gender inequality [78]. In light of this, can biological frameworks offer support?

Evolutionary anthropologists pay much attention to physical sex differences that relate to reproduction [79–81]. Two quintessential traits of our genus—bipedal locomotion and encephalized brains—uniquely impose on females both the risk of birth injury and the direct metabolic costs of nourishing the foetus and infant [77]. But since reproduction benefits both sexes, these physical costs present zero justification for societal gender inequality, so why is it so common?

The P–S game offers a fresh perspective on gender dynamics, and how inequalities might be lessened by changing societal norms. My aim here is not to contradict other approaches, but to consider how insights from game theory can support their efforts.

In sexually reproducing organisms, it has long been recognized that males and females ‘play a game’ around the time of conception, determining how the costs of parental care are distributed [82]. In teleost fish, with external fertilization, females can escape once they have provided ova for fertilization, and males are the obligatory providers of care. In birds and mammals, where fertilization is internal, females are left ‘holding the baby’ [82], though males may still help. This asymmetry has implications for P–S dynamics, and mammalian male activities can be interpreted in terms of a ‘scrounger pay-off’ [18].

In mammals, the unique female contribution to pregnancy and lactation makes these energy-rich investments prone to control and exploitation by males. Females may ring-fence energy within the body (fat stores) during pregnancy, in order to prevent the resources required for lactation from being scrounged by others, including males [83]. However, successive offspring can create maternal energy-debt, placing mothers at unique risk of undernutrition (‘maternal depletion’) [84]. In addition to these direct costs, norms of gender inequality, typically based on ideals of women as ‘reproducers’, often lead to their being allocated low-status activities, including unpaid care work in the household and mundane subsistence tasks [85]. The savings that males make on such activities can be reallocated to male–male competition, or to reproduction with other females. These male benefits are essentially funded by scrounging the energy costs of reproduction, childcare and subsistence from women [18].

Gender inequality has pervasive costs for women’s health, especially maternal health [86, 87], and also correlates with high levels of child undernutrition in the next generation [88]. Gender inequality thus undermines the health and fitness of both sexes. Despite these penalties, the problem has an inherent intransigence: from an evolutionary perspective, the social strategies of individual males are not under selective pressure to optimize health, but rather to outcompete other males for fitness.

However, these dynamics can be altered by efforts to change societal norms. Movements for gender equality have made substantial progress in many countries, though much remains to be done. Societal norms can profoundly shift P–S dynamics, ‘renegotiating’ the level of scrounging by males. Amongst the targets have been:

- Preventing early marriage to increase female autonomy over reproductive scheduling, which benefits both maternal and child health [89–91]
- Supporting breastfeeding to reduce conflict between women’s reproductive and productive roles [92, 93]
- Promoting girl’s education, to promote empowerment, improve access to paid labour and reduce friction in marital/similar relationships [94, 95]
- Providing maternity leave and improving job security, which improves maternal physical and mental health and reduces the economic penalty for reproduction [96]
- Promoting norms for paternal care of children (e.g. paternity leave), which improves maternal mental and physical health and supports breastfeeding [97]

All of these activities indicate active societal renegotiations, to reduce the magnitude of male scrounging by challenging the social institutions that legitimize it. Interventions that target gender norms have had mixed success [98], making this a continuing research priority.

## SOCIO-ECONOMIC INEQUALITY WITHIN AND BETWEEN POPULATIONS

Dominance hierarchies are found in many species including primates [23, 99, 100]. The key difference in humans is the way that dominants operationalize scrounging, by actively coercing labour and resources from producers, and institutionalizing this process through the use of values, norms, laws and practices as well as physical forms of coercion. For most of history, the P–S game has played out broadly similarly across diverse kinds of society. This framework offers a clear answer to the question, *why* are human societies so prone to hierarchy?

Only limited forms of hierarchy can usually emerge in societies practicing hunting and gathering or simple horticulture, because the physical effort of each individual limits productivity [101], and minimizes the potential returns from scrounging (though in a few sedentary hunter–gatherer societies, productivity was enough to allow persistent inequality [28, 102]). Moreover, hunter–gatherer societies often apply ‘levelling’ mechanisms to prevent any individual from gaining status and power [103]. Tolerated scrounging, through which individuals in need can ask for help, is a mutually-beneficial survival strategy for the whole population, given that individuals’ foraging returns are unpredictable [29].

However, as soon as agricultural systems raised productivity, the pay-off for scrounging likewise rose. A key threshold was reached when the plough and irrigation systems increased food output per unit of land, and animals underwent domestication [104, 105]. Not only did intensive farming thereby increase total food availability, but it also packaged it into forms ideal for scrounging: animal flocks, haystacks and granaries [18].

Early agricultural societies thus became prone to two generic forms of scrounging: first, taxation by internal elites (landlords) who secured ownership of the land and forced tenant farmers (peasants) to hand over part of the harvest as rent [106]; and second, external aggressors, often nomadic horsemen who raided the harvests [107]. A key underlying mechanism was the concept of debt [108], through which those who had borrowed resources and could not repay were forced to surrender their land-rights [106], and become a captive stock of producers [18, 109]. Through history, elites of agrarian societies have favoured grain agriculture, as the harvests are ideal for storage and taxation; this has driven a global shift since the emergence of intensive agriculture to diets high in carbohydrate [110].

The basic ‘landlord-peasant’ version of the P–S game has persisted in different forms through the history of agriculture, and can be discerned in many contemporary societies globally. In all such societies, producers are forced in different ways to feed the elites, whilst also reproducing themselves. Producers might be physically coerced, the most obvious example being forms of slavery [111–113]. In most systems, however, the ultimate driver of work was hunger, combined with the lack of opportunity for producers to opt out of this system. Scrounger groups make extensive efforts to prevent their producers from leaving. In this scenario, if producers do not farm they will starve.

The key issue for peasants is not how much of the harvest is scrounged, but how much they are left with to live on [109]—the finder’s share. Excessive levels of scrounging could make the entire social order unviable. When in control of their producers, landlords can maintain a viable social order either by increasing the finder’s share to incentivize production, or by coercing the producers to work harder, leading towards a system of slavery (Supplementary Fig. S1). The two parties may negotiate over this dilemma. For example, Scott has described how, in 19th century South-East Asian societies, the stress of famine prompted peasants to rebel, but not to cast off the landlords altogether, rather to demand greater support from them during famines [109]—effectively, a ‘refund’ of the finder’s share. In extreme conditions, producers may quit the system altogether: there are records from many historical periods of peasants returning to marginal environments, to practice forms of immediate-return subsistence that are relatively immune to scrounging [114]. However, due to institutions of land ownership, voting with one’s feet has often not been possible. Until recently, only rarely did producers amass sufficient power to reduce and challenge their exploitation—either through developing democratic institutions, or through outright revolution [115]. Moreover, whenever such a societal shift is achieved new groups of scroungers may rapidly emerge, operationalizing new forms of power over producers.

In 19th century colonialism, entire countries were co-opted to produce under the control of imperial scroungers [18]. As expected, this led not only to the international movement of resources, but also to the divergence of health outcomes, whose ramifications are still being felt today. For example, approximately one fifth of wheat consumed in the industrializing UK in the late 19th century was provided by imports from India, even as a series of famines caused devastating malnutrition in India [116]. Globally, the legal, coercive and military apparatus of colonialism maintained this international version of the P–S game amongst hundreds of millions of people.

## INCORPORATING CONSUMPTION

In recent centuries, the P–S game has undergone a unique shift, with profound implications for the nature and distribution of health inequalities. This occurred first within high-income industrialized countries, driven by the consolidation of capitalist economics, and more recently globally.

**Figure 3.**
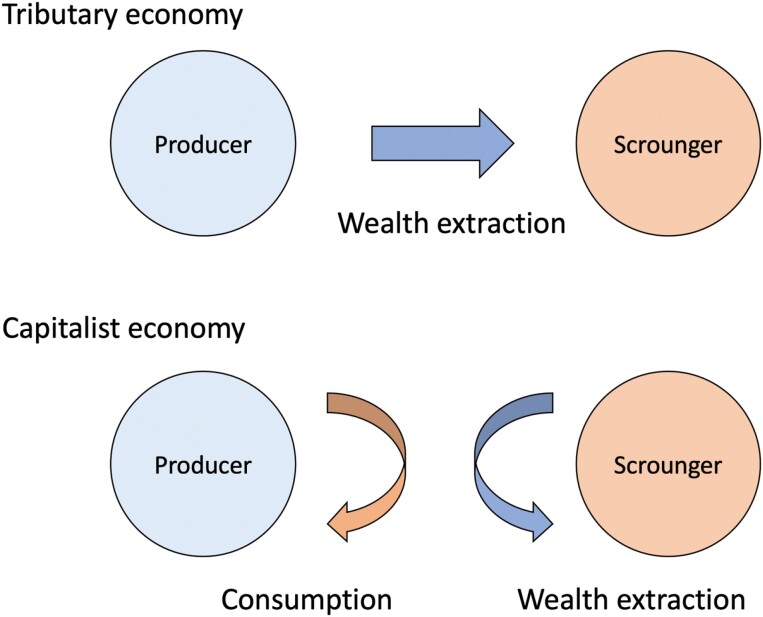
The logic of wealth extraction in tributary and capitalist societies. (a) In tributary societies, food and other raw resources are extracted from producers, and disproportionately consumed by scroungers. Scroungers become wealthy by amassing more resources (b) In capitalist societies, producers, especially in urban industrial environments, must purchase their food. Scroungers now extract wealth by using markets to exert control over both production and consumption. Reproduced with permission from Wells, 2016 [18].

Up until a point, scrounger-elites amassed resources directly from producers. Consuming more of these resources was the primary pay-off from scrounging. Under the logic of industrial capitalism, however, scroungers began to seek wealth rather than specific resources [117, 118], and wealth generation required increasing the numbers of consumers. Capitalist economics thus saw producers fundamentally re-fashioned as consumers (**Fig. 3**). This reorganization was both embedded in, and has driven, a wave of nutrition transitions [119, 120] occurring at different times in different regions, as producers changed from farmers to wage earners, and industrially manufactured foods transformed diets [113, 121, 122].

Several elements of global food systems have changed in concert. First, increasing numbers of food producers have been driven off the land, transitioning to urban–industrial work and purchasing their food through markets. Second, new diets have emerged in which foods are tailored to maximize consumption, though often at a cost to health [123]. Through the 19th–20th centuries sugar consumption increased exponentially [113], while attention now focuses on ultra-processed foods [120]. Both of these dietary components have been linked with obesity and the risk of non-communicable disease [124, 125]. A significant proportion of global agriculture produces products that are quintessentially addictive (tobacco, tea, coffee) and highly profitable, and yet contribute negligibly to meeting dietary energy requirements [126]. Sales and advertising strategies drive up consumption, but the financial costs are ultimately paid by the consumers through their purchasing, emphasizing once again how scroungers fund their exploitation from the effort of others.

This scenario underpins ongoing nutrition transition today in low- and middle-income countries (LMICs). The emerging ‘double burden of malnutrition’ acutely illustrates how LMIC populations have been captured in both old and new versions of the P–S game [18]. As food producers, LMIC populations have long been prone to childhood undernutrition [127]. As new consumers, the same populations are increasingly prone to overweight [122]. Life-course exposure to the double burden, highlighted by secular increases in body mass index but not height [128], represents a fast track to non-communicable disease [129].

## RACISM AND OTHERING

Hierarchies can emerge in many species, but a unique aspect in humans is the use of coercion to drive unequal P–S pay-offs. Physical coercion has undoubtedly played a key role, as has the threat of hunger and starvation for producers who cannot opt out [14]. However, violence is potentially costly for all parties, and is difficult to operationalize in societies with thousands or millions of producers. Another fundamental mechanism is the use of ideology, generating social norms that symbolically assign low status to producers and reify the unequal social order.

In early agricultural societies, it was no coincidence that early elites often held office in temples, allowing the development of religious ideologies that justified the status quo [106]. Early emerging contrasts were legally defined statuses such as slave versus free person, landlord versus tenant, or caste groups [111, 112, 115]. Over time, an ever-increasing range of categorical distinctions have emerged, with a common theme of disempowering producers to prevent them from challenging the ideological system that exploits them.

Crucially, this approach provides an overarching perspective on racism. Through history, the creation of racialized societies and similarly institutionalized forms of inequality has functioned to create and maintain sources of cheap producer–labour. Common to plantation slavery in the New World, apartheid in South Africa and the Indian caste system was the long-term maintenance of a workforce that could be coerced through both physical and symbolic means to produce under highly asymmetric conditions [111, 130, 131].

Some forms of hierarchy allow individuals to change their status, such as slaves earning free status or tenants earning enough to become landowners. In contrast, racial and caste identities externally assign individuals to identities from which they cannot opt out [132], thus hindering them from challenging their role in the social system. I used the term ‘metabolic ghetto’ specifically to highlight how the dominant groups in racialized societies use coercion, symbolic categories and social norms to maintain power over other groups, shaping their labour, their access to resources and their living conditions. Persisting major inequalities in physical and mental health in racialized societies show the pernicious effect of these categorizations [2, 133], highlighting the need for broad, deep and transformative action to challenge the drivers and determinants of racism and discrimination [134]. A complementary approach is the recent exploration of racialized societies through the lens of niche construction by Henry *et al*. [38].

**Figure 4.**
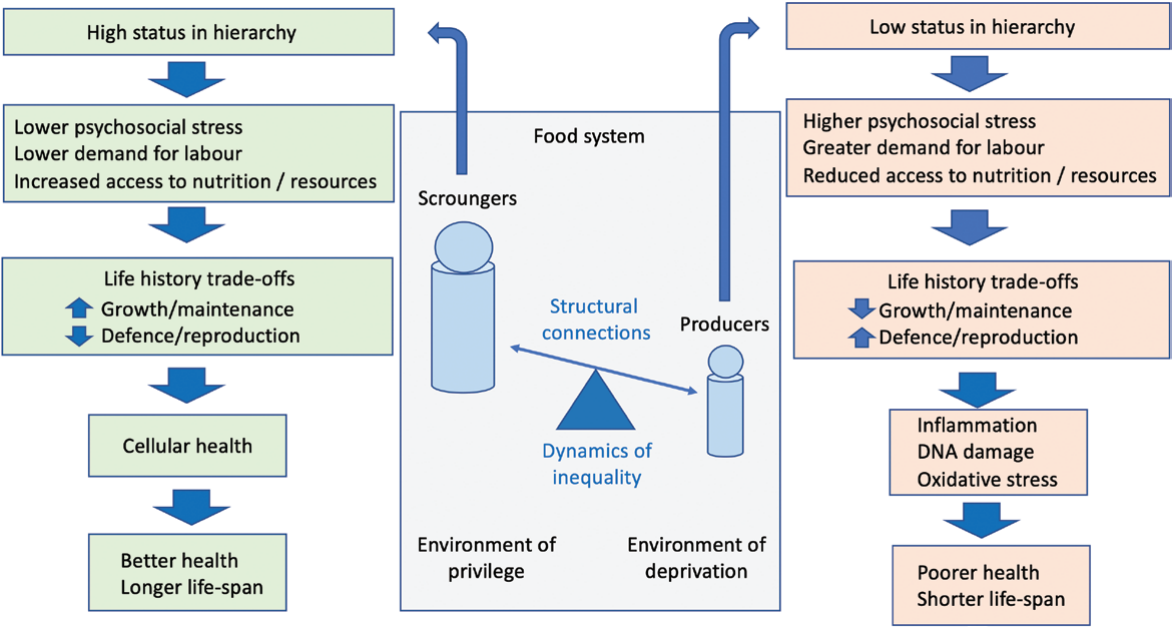
Schematic diagram illustrating how racialized societies involve producers and scroungers occupying environments of contrasting quality, shaping patterns of both production (work) and consumption (e.g. diet), thereby driving contrasting life history trade-offs that underpin health inequalities.

Violence and ideology do not merely maintain disproportionate control over the production and consumption of resources; by chronically activating the stress response, violence or its threat also reduces the allocation of metabolic resources to homeostasis [135, 136], undermining the growth and health of producers over inter-generational timescales [2]. Health disparities carry the collective imprints of different workloads, differential nutrition and environmental stresses that shape contrasting energy-allocation trade-offs (**Fig. 4**). While violence and physical injury might seem the severest threats, the ideological component of P–S hierarchies must be recognized as being more effective for scroungers, in part because ideological hierarchies conceal their true costs inside forms of disease that develop cumulatively through the life course [18].

## CONCLUSION

This article offers an over-arching framework, based on dynamic game theory, that can help understand how diverse forms of social inequality generate health disparities. In contrast to other evolutionary perspectives that focus on the ecological characteristics of food resources [137], my framework focuses explicitly on relationships of exploitation among people, and considers why some humans often seek to control, ideologically or physically, others using different forms of power. The founders of both modern economics and archaeology made frequent reference to the concept of ‘surplus’ generated by agriculture, whose redistribution transformed human societies [138, 139]. The P–S game offers a lens through which to ask how freely this surplus is generated and distributed [18]. In fact, the emergence of ‘surplus’ did not require agriculture: slavery appears to have been practiced by some prehistorical hunter–gatherers occupying resource-rich settings [28, 140], and the practice persists today even in high-income countries, where unsurprisingly its manifestation is closely associated with the production of food and other raw materials [141, 142].

In common with other theoretical frameworks, this evolutionary perspective is intended to help understand, but not to justify, forms of social inequality and exploitation. ‘*The Metabolic Ghetto*’ explored in detail how P–S dynamics have emerged and varied across societies, over historical time and across different economic and political systems [18], focussing in particular on the role of capitalist economics in the emerging global epidemic of non-communicable disease. The P–S game fits well with life history theory, thus providing a comprehensive evolutionary framework for understanding how social inequalities drive the life-course emergence of health disparities. The model could be made more sophisticated, for example, modelling social gradients rather than discrete groups.

In turn, this approach may offer insight into potential interventions for reducing disparities, through renegotiating unequal P–S pay-offs. Ultimately, reducing health inequalities requires not only distributing resources and opportunities more equally but also suppressing exploitation by changing social institutions. Specific tactics could involve targeting tangible markers of the parameters modelled in the game, such as the ‘rate of searching/producing’, the magnitude of the ‘finder’s share’ or the effective size of the group, but also challenging the vested interests and social norms that maintain unfavourable parameters. That the P–S game is increasingly used by both ecologists and economists [143] suggests that it has unique potential to shed new light on the societal basis of health inequalities.

## Supplementary Material

eoad026_suppl_Supplementary_Appendix

## References

[1] Marmot M. Social determinants of health inequalities. Lancet 2005;365:1099–104. doi: 10.1016/S0140-6736(05)71146-6. 15781105

[2] Selvarajah S , Corona Maioli S, Deivanayagam TA et al. Racism, xenophobia, and discrimination: mapping pathways to health outcomes. Lancet 2022;400:2109–24. doi: 10.1016/S0140-6736(22)02484-9. 36502849

[3] Winslow C. The untilled fields of public health. Science 1920;51:23–33. 17838891 10.1126/science.51.1306.23

[4] Lagarde M , Haines A, Palmer N. Conditional cash transfers for improving uptake of health interventions in low- and middle-income countries: a systematic review. JAMA 2007;298:1900–10. doi: 10.1001/jama.298.16.1900. 17954541

[5] Fernald LC , Gertler PJ, Neufeld LM. Role of cash in conditional cash transfer programmes for child health, growth, and development: an analysis of Mexico’s Oportunidades. Lancet 2008;371:828–37. doi: 10.1016/S0140-6736(08)60382-7. 18328930 PMC2779574

[6] Rezaei SJ , de Walque D, Mateen FJ. Conditional cash transfers to improve health-focused outcomes: a global scoping review. Glob Public Health 2022;17:3368–85. doi: 10.1080/17441692.2022.2092186. 35727705

[7] Watts P , Buck D, Netuveli G, Renton A. Clustering of lifestyle risk behaviours among residents of forty deprived neighbourhoods in London: lessons for targeting public health interventions. J Public Health (Oxf) 2016;38:308–15. doi: 10.1093/pubmed/fdv028. 25762701 PMC4894479

[8] Vissenberg C , Nierkens V, van Valkengoed I et al. The impact of a social network based intervention on self-management behaviours among patients with type 2 diabetes living in socioeconomically deprived neighbourhoods: a mixed methods approach. Scand J Public Health 2017;45:569–83. doi: 10.1177/1403494817701565. 28707567 PMC5544123

[9] Sellstrom E , Arnoldsson G, Alricsson M, Hjern A. Obesity prevalence in a cohort of women in early pregnancy from a neighbourhood perspective. BMC Pregnancy Childbirth 2009;9:37. doi: 10.1186/1471-2393-9-37. 19706158 PMC2744903

[10] Amegah AK , Damptey OK, Sarpong GA et al. Malaria infection, poor nutrition and indoor air pollution mediate socioeconomic differences in adverse pregnancy outcomes in Cape Coast, Ghana. PLoS One 2013;8:e69181. doi: 10.1371/journal.pone.0069181. 23894428 PMC3718681

[11] Devakumar D , Selvarajah S, Shannon G et al. Racism, the public health crisis we can no longer ignore. Lancet 2020;395:e112–3. doi: 10.1016/S0140-6736(20)31371-4. 32534630 PMC7289562

[12] World Health Organization. Global, Regional and National Estimates for Intimate Partner violence Against Women and Global and Regional Estimates for Non-partner Sexual Violence Against Women. Geneva: World Health Organization, on behalf of the United Nations Inter-Agency Working Group on Violence Against Women Estimation and Data (UNICEF, UNFPA, UNODC, UNSD, UNWomen), 2021.

[13] Gradin C , Leibbrandt M, Tarp F. Inequality in the Developing World. Oxford: Oxford University Press, 2021.

[14] Townsend J. A Dissertation on the Poor Laws by a Well-wisher to Mankind. Berkeley, CA: University of California Press, 1786/1971.

[15] Stuckler D , Reeves A, Loopstra R et al. Austerity and health: the impact in the UK and Europe. Eur J Public Health 2017;27:18–21. doi: 10.1093/eurpub/ckx167. 29028245 PMC5881725

[16] Grubb A , Turner E. Attribution of blame in rape cases: a review of the impact of rape myth acceptance, gender role conformity and substance use on victim blaming. Aggression and Violent Behavior 2012;17:443–52.

[17] Chagnon NG. Racialized culpability: victim blaming and state violence. Race Ethnicity and Law 2017;22:199–219.

[18] Wells JC. The Metabolic Ghetto: An Evolutionary Perspective on Nutrition, Power Relations and Chronic Disease. Cambridge: Cambridge University Press, 2016.

[19] Sasaki T , Penick CA, Shaffer Z et al. A simple behavioral model predicts the emergence of complex animal hierarchies. Am Nat 2016;187:765–75. doi: 10.1086/686259. 27172595

[20] Chase ID , Tovey C, Spangler-Martin D, Manfredonia M. Individual differences versus social dynamics in the formation of animal dominance hierarchies. Proc Natl Acad Sci USA 2002;99:5744–9. doi: 10.1073/pnas.082104199. 11960030 PMC122842

[21] Hobson EA. Differences in social information are critical to understanding aggressive behavior in animal dominance hierarchies. Curr Opin Psychol 2020;33:209–15. doi: 10.1016/j.copsyc.2019.09.010. 31627042

[22] Sapolsky RM. The influence of social hierarchy on primate health. Science 2005;308:648–52. 15860617 10.1126/science.1106477

[23] Franz M , McLean E, Tung J et al. Self-organizing dominance hierarchies in a wild primate population. Proc Biol Sci 2015;282:20151512. doi: 10.1098/rspb.2015.1512. PMC4571707 26336168

[24] Boyd R , Richerson PJ. Culture and the evolution of human cooperation. Philos Trans R Soc Lond B Biol Sci 2009;364:3281–8. doi: 10.1098/rstb.2009.0134. 19805434 PMC2781880

[25] Henrich J , Muthukrishna M. The origins and psychology of human cooperation. Annu Rev Psychol 2021;72:207–40. doi: 10.1146/annurev-psych-081920-042106. 33006924

[26] Apicella CL , Silk JB. The evolution of human cooperation. Curr Biol 2019;29:R447–50. doi: 10.1016/j.cub.2019.03.036. 31163155

[27] Apicella CL , Marlowe FW, Fowler JH, Christakis NA. Social networks and cooperation in hunter-gatherers. Nature 2012;481:497–501. doi: 10.1038/nature10736. 22281599 PMC3340565

[28] Kelly RL. The Foraging Spectrum. Washington: Smithsonian Institution Press, 1995.

[29] Howell N. Life Histories of the Dobe!Kung: Food, Fatness, and Well-being Over the Life-span. Berkeley: University of California Press, 2010.

[30] Wiessner P , Schiefenhovel W. Food and the Status Quest: An Interdisciplinary Perspective. Providence, RI: Berghahn Books, 1997.

[31] Wilkinson R , Pickett K. The Spirit Level: Why More Equal Societies Almost Always Do Better. London: Allen Lane, 2009.

[32] LaVeist TA , Gaskin D, Richard P. Estimating the economic burden of racial health inequalities in the United States. Int J Health Serv 2011;41:231–8. doi: 10.2190/HS.41.2.c. 21563622

[33] Odling-Smee FJ , Laland K, Feldman MW. Niche Construction. Princeton: Princeton University Press, 2003.

[34] Laland KN , Odling-Smee J, Myles S. How culture shaped the human genome: bringing genetics and the human sciences together. Nat Rev Genet 2010;11:137–48. 20084086 10.1038/nrg2734

[35] Shennan S. Property and wealth inequality as cultural niche construction. Philos Trans R Soc Lond B Biol Sci 2011;366:918–26. doi: 10.1098/rstb.2010.0309. 21320904 PMC3048998

[36] Boehm C , Flack JC; The emergence of simple and complex power structures through social niche construction. In: Guinote A, Vescio TKS (eds). The social psychology of power. New York: The Guilford Press, 2010, 46–6.

[37] Fuentes A ; Cooperation, conflict and niche construction in the Genus Homo. In: Fry DPS (ed). War, Peace, and Human Nature: The Convergence of Evolutionary and Cultural Views. Oxford: Oxford University Press, 2013, 78–94.

[38] Henry PI , Beaulieu MRS, Bradford A et al. Embedded racism: inequitable niche construction as a neglected evolutionary process affecting health. Evolution Medicine and Public Health 2023;11:112–125. 37197590 10.1093/emph/eoad007 PMC10184440

[39] Lehmann L. The adaptive dynamics of niche constructing traits in spatially subdivided populations: evolving posthumous extended phenotypes. Evolution 2008;62:549–66. 17983464 10.1111/j.1558-5646.2007.00291.x

[40] Barnard CJ , Sibly RM. Producers and scroungers: a general model and its application to captive flocks of house sparrows. Anim Behav 1981;29:543–50.

[41] Vickery WL , Giraldeau L-A, Templeton JJ et al. Producers, scroungers, and group foraging. Am Nat 1991;137:847–63.

[42] Morand-Ferron J , Giraldeau L-A, Lefebvre L. Wild Carib grackles play a producer–scrounger game. Behav Ecol 2007;18:916–21.

[43] Barta Z ; Producer-scrounger models and aspects of natural resource use. In: Giraldeau L-AS (ed). Investors and Exploiters in Ecology and Economics: Principles and Applications. Cambridge, MA: MIT Press, 2017, 65–82.

[44] Steele WK , Hockey PA. Factors influencing rate and success of intraspecific kleptoparasitism among Kelp Gulls (*Larus dominicanus*). Auk 1995;112:847–59.

[45] Brockmann HJ , Barnard CJ. Kleptoparasitism in birds. Anim Behav 1979;27:487–514.

[46] Hansen AJ. Fighting behavior in Bald Eagles: a test of game theory. Ecol 1986;67:787–97.

[47] King AJ , Isaac NJ, Cowlishaw G. Ecological, social and reproductive factors shape producer-scrounger dynamics in baboons. Behav Ecol 2009;20:1039–49.

[48] Lee AEG , Cowlishaw G. Switching spatial scale reveals dominance-dependent social foraging tactics in a wild primate. PeerJ 2017;5:e3462. doi: 10.7717/peerj.3462. 28674647 PMC5494171

[49] Barta Z , Giraldeau L-A. The effect of dominance hierarchy on the use of alternative foraging tactics: a phenotype-limited producing-scrounging game. Behav Ecol Sociobiol 1998;42:217–23.

[50] McCormack JE , Jablonski PG, Brown JL. Producer-scrounger roles and joining based on dominance in a free-living group of Mexican jays (*Aphelocoma ultramarina*). Behav 2007;144:967–82.

[51] Wolf M , van Doorn GS, Leimar O, Weissing FJ. Life-history trade-offs favour the evolution of animal personalities. Nature 2007;447:581–4. doi: 10.1038/nature05835. 17538618

[52] Hamilton IM , Benincasa MD. Emergence of size-structured dominance hierarchies through size-dependent feedback. Philos Trans R Soc Lond B Biol Sci 2022;377:20200449. doi: 10.1098/rstb.2020.0449. 35000447 PMC8743889

[53] Watve MG , Yajnik CS. Evolutionary origins of insulin resistance: a behavioral switch hypothesis. BMC Evol Biol 2007;7:61. 17437648 10.1186/1471-2148-7-61 PMC1868084

[54] Phillips JA , Peacock SJ, Bateman A et al. An asymmetric producer-scrounger game: body size and the social foraging behavior of coho salmon. Theor Ecol 2018;11:417–31. doi: 10.1007/s12080-018-0375-2. 30931016 PMC6405016

[55] Hausfater G , Altmann J, Altmann S. Long-term consistency of dominance relations among female baboons (Papio cynocephalus). Science 1982;217:752–5. doi: 10.1126/science.217.4561.752. 17772319

[56] Foerster S , Franz M, Murray CM et al. Chimpanzee females queue but males compete for social status. Sci Rep 2016;6:35404. doi: 10.1038/srep35404. 27739527 PMC5064376

[57] Stearns SC. The Evolution of Life Histories. Oxford: Oxford University Press, 1992.

[58] Wells JCK , Nesse RM, Sear R et al. Evolutionary public health: introducing the concept. Lancet 2017;390:500–9. doi: 10.1016/S0140-6736(17)30572-X. 28792412

[59] Watve M. Doves, Diplomats and Diabetes: A Darwinian Interpretation of Type 2 Diabetes and Related Disorders. New York: Springer, 2013.

[60] Kim D , Saada A. The social determinants of infant mortality and birth outcomes in Western developed nations: a cross-country systematic review. Int J Environ Res Public Health 2013;10:2296–335. doi: 10.3390/ijerph10062296. 23739649 PMC3717738

[61] Hajizadeh M , Nandi A, Heymann J. Social inequality in infant mortality: what explains variation across low and middle income countries? Soc Sci Med 2014;101:36–46. doi: 10.1016/j.socscimed.2013.11.019. 24560222

[62] Victora CG , Barros FC, Vaughan JP et al. Birthweight, socio-economic status and growth of Brazilian infants. Ann Hum Biol 1987;14:49–57. 3592612 10.1080/03014468700008831

[63] Spencer N , Bambang S, Logan S, Gill L. Socioeconomic status and birth weight: comparison of an area-based measure with the Registrar General’s social class. J Epidemiol Community Health 1999;53:495–8. 10562868 10.1136/jech.53.8.495 PMC1756936

[64] Geronimus AT. Black/white differences in the relationship of maternal age to birthweight: a population-based test of the weathering hypothesis. Soc Sci Med 1996;42:589–97. 8643983 10.1016/0277-9536(95)00159-x

[65] van Soest A , Saha UR. Relationships between infant mortality, birth spacing and fertility in Matlab, Bangladesh. PLoS One 2018;13:e0195940. doi: 10.1371/journal.pone.0195940. 29702692 PMC5922575

[66] Bradshaw CJA , Perry C, Judge MA et al. Lower infant mortality, higher household size, and more access to contraception reduce fertility in low- and middle-income nations. PLoS One 2023;18:e0280260. doi: 10.1371/journal.pone.0280260. 36812163 PMC9946217

[67] Hamlat EJ , Adler NE, Laraia B et al. Association of subjective social status with epigenetic aging among Black and White women. Psychoneuroendocrinology 2022;141:105748. doi: 10.1016/j.psyneuen.2022.105748. 35397259 PMC10228718

[68] Noren Hooten N , Pacheco NL, Smith JT, Evans MK. The accelerated aging phenotype: The role of race and social determinants of health on aging. Ageing Res Rev 2022;73:101536. doi: 10.1016/j.arr.2021.101536. 34883202 PMC10862389

[69] Wells JC. Maternal capital and the metabolic ghetto: An evolutionary perspective on the transgenerational basis of health inequalities. Am J Hum Biol 2010;22:1–17. 19844897 10.1002/ajhb.20994

[70] Anderson JA , Johnston RA, Lea AJ et al. High social status males experience accelerated epigenetic aging in wild baboons. Elife 2021;10:e66128. doi: 10.7554/eLife.66128. 33821798 PMC8087445

[71] Opondo C , Gray R, Hollowell J et al. Joint contribution of socioeconomic circumstances and ethnic group to variations in preterm birth, neonatal mortality and infant mortality in England and Wales: a population-based retrospective cohort study using routine data from 2006 to 2012. BMJ Open 2019;9:e028227. doi: 10.1136/bmjopen-2018-028227. PMC6677942 31371291

[72] Orr J , Freer J, Morris JK et al. Regional differences in short stature in England between 2006 and 2019: a cross-sectional analysis from the National Child Measurement Programme. PLoS Med 2021;18:e1003760. doi: 10.1371/journal.pmed.1003760. 34582440 PMC8478195

[73] Census. 2021. Health state life expectancies by national deprivation deciles. Office for National Statistics.

[74] Wells JCK , Cole TJ, Cortina-Borja M et al. Low maternal capital predicts life history trade-offs in daughters: why adverse outcomes cluster in individuals. Front Public Health 2019;7:206. doi: 10.3389/fpubh.2019.00206. 31417889 PMC6685417

[75] Wells JCK , Cole TJ, Cortina-Borja M et al. Life history trade-offs associated with exposure to low maternal capital are different in sons compared to daughters: evidence from a prospective Brazilian birth cohort. Front Public Health 2022;10:914965. doi: 10.3389/fpubh.2022.914965. 36203666 PMC9532015

[76] Wells JC ; The impact of social dynamics on life history trajectory and demographic traits: insights from the ‘producer-scrounger’ game. In: Burger O, Lee R, Sear RS (eds). Human Evolutionary Demography. Cambridge: Open Book Publishers, 2023. In press.

[77] Huber J. On the Origins of Gender Inequality. Boulder, CO: Paradigm Publishers, 2007.

[78] Di Leonardo M. Gender at the Crossroads: Feminist Anthropology in Historical Perspective. Berkely: University of California Press, 1991.

[79] Bribiescas RG. Reproductive ecology and life history of the human male. Am J Phys Anthropol 2001;Suppl 33:148–76. doi: 10.1002/ajpa.10025.abs. 11786994

[80] Vitzthum VJ. The ecology and evolutionary endocrinology of reproduction in the human female. Am J Phys Anthropol 2009;140:95–136. doi: 10.1002/ajpa.21195. 19890865

[81] Wells JC. Sexual dimorphism in body composition across human populations: associations with climate and proxies for short- and long-term energy supply. Am J Hum Biol 2012;24:411–9. doi: 10.1002/ajhb.22223. 22345077

[82] Dawkins R , Carlisle TR. Parental investment, mate desertion and a fallacy. Nature 1976;262:131–3.

[83] Wells JC. The capital economy in hominin evolution: how adipose tissue and social relationships confer phenotypic flexibility and resilience in stochastic environments. Curr Anthropol 2012;53(Suppl. 6):466–78.

[84] Kozuki N , Walker N. Exploring the association between short/long preceding birth intervals and child mortality: using reference birth interval children of the same mother as comparison. BMC Public Health 2013;13:S6. doi: 10.1186/1471-2458-13-S3-S6. PMC3847658 24564713

[85] Marphatia AA , Moussié R. A question of gender justice: exploring the linkages between women’s unpaid care work, education, and gender equality. Int J Educ Res 2013;33:585–94.

[86] Moss NE. Gender equity and socioeconomic inequality: a framework for the patterning of women’s health. Soc Sci Med 2002;54:649–61. doi: 10.1016/s0277-9536(01)00115-0. 11999484

[87] Grown C , Gupta GR, Pande R. Taking action to improve women’s health through gender equality and women’s empowerment. Lancet 2005;365:541–3. doi: 10.1016/S0140-6736(05)17872-6. 15705464

[88] Marphatia AA , Cole TJ, Grijalva-Eternod CS, Wells JCK. Associations of gender inequality with child malnutrition and mortality across 96 countries. Global Health Epidemiol. Genom. 2016;1:e6. 10.1017/gheg.2016.1 PMC5870432 29868199

[89] Marphatia AA , Ambale GS, Reid AM. Women’s marriage age matters for public health: a review of the broader health and social implications in South Asia. Front Public Health 2017;5:269. doi: 10.3389/fpubh.2017.00269. 29094035 PMC5651255

[90] Wells JCK. An evolutionary model of ‘sexual conflict’ over women’s age at marriage: implications for child mortality and undernutrition. Front Public Health 2022;10:653433. doi: 10.3389/fpubh.2022.653433. 35784199 PMC9247288

[91] Marphatia AA , Saville NM, Manandhar DS et al. Independent associations of women’s age at marriage and first pregnancy with their height in rural lowland Nepal. Am J Phys Anthropol 2021;174:103–16. doi: 10.1002/ajpa.24168. 33166434

[92] Van Esterik P. Breastfeeding and feminism. Int J Gynaecol Obstet 1994;47(Suppl):S41–50; discussion S50. doi: 10.1016/0020-7292(94)02233-o. 7713306

[93] Labbok MH , Smith PH, Taylor EC. Breastfeeding and feminism: a focus on reproductive health, rights and justice. Int Breastfeed J 2008;3:8. doi: 10.1186/1746-4358-3-8. 18680575 PMC2531083

[94] Le K , Nguyen M. How education empowers women in developing countries. B.E. Journal of Economic Analysis and Policy 2021;21:511–36.

[95] Marcus R , Page E. Girl’s Learning and Empowerment - the Role of School Environments. United Nations Girls’ Education Initiative, 2016.

[96] Staehelin K , Bertea PC, Stutz EZ. Length of maternity leave and health of mother and child--a review. Int J Public Health 2007;52:202–9. doi: 10.1007/s00038-007-5122-1. 18030952

[97] Van Niel MS , Bhatia R, Riano NS et al. The impact of paid maternity leave on the mental and physical health of mothers and children: a review of the literature and policy implications. Harv Rev Psychiatry 2020;28:113–26. doi: 10.1097/HRP.0000000000000246. 32134836

[98] Kraft JM , Wilkins KG, Morales GJ et al. An evidence review of gender-integrated interventions in reproductive and maternal-child health. J Health Commun 2014;19(Suppl 1):122–41. doi: 10.1080/10810730.2014.918216. 25207450 PMC4205884

[99] Cowlishaw G , Dunbar RIM. Dominance rank and mating success in male primates. Anim Behav 1991;41:1045–56.

[100] Pusey A , Williams J, Goodall J. The influence of dominance rank on the reproductive success of female chimpanzees. Science 1997;277:828–31. 9242614 10.1126/science.277.5327.828

[101] Smith EA , Hill K, Marlowe FW et al. Wealth transmission and inequality among hunter-gatherers. Curr Anthropol 2010;51:19–34. 21151711 10.1086/648530 PMC2999363

[102] Hayden B ; Richman, poorman, beggarman, chief: the dynamics of social inequality. In: Feinman G, Price TDS (eds). Archaeology at the Millennium: A Sourcebook. New York: Springer, 2001, 231–272.

[103] Wiessner P. Levelling the hunter: constraints on the social quest in foraging soceties. In: Wiessner P, Schiefenhovel WS (eds). Food and the Status Quest: An Interdisciplinary Perspective. Providence, RI: Bergahn Books, 1997, 171–191.

[104] Gurven M , Borgerhoof Mulder M, Hooper PL et al. Domestication alone does not lead to inequality: intergenerational wealth transmission among horticulturalists. Curr Anthropol 2010;51:49–64.

[105] Shenk MK , Borgerhoff Mulder M, Beise J et al. Intergenerational wealth transmission among agriculturalists: foundations of agrarian inequality. Curr Anthropol 2010;51:65–83.

[106] Graeber D. Debt: the First 5,000 Years. London: Melville House, 2011.

[107] McNeill W. Plagues and Peoples. New York: Doubleday, 1977.

[108] Ingham G. The Nature of Money. Cambridge: Polity Press, 2004.

[109] Scott J. The Moral Economy of the Peasant: Rebellion and Subsistence in Southeast Asia. New Haven: Yale University Press, 1976.

[110] Scott JC. Against the Grain: A Deep History of the Earliest States. New Haven, CT: Yale University Press, 2017.

[111] Chakravarti U. Of Dasas and Karmakaras: servile labour in ancient India. In: Patnaik U, Dingwaney MS (eds). Chains of Servitude: Bondage and Slavery in India. Madras: Sangam Books Ltd, 1985, 35–75.

[112] Flannery K , Joyce M. The Creation of Inequality: How Our Prehistoric Ancestors Set the Stage for Monarchy, Slavery, and Empire. Cambridge, MA: Harvard University Press, 2012.

[113] Mintz SW. Sweetness and Power: The Place of Sugar in Modern History. New York: Viking Penguin Inc, 1985.

[114] Scott JC. The Art of Not Being Governed: An Anarchist History of Upland Southeast Asia. Newhaven, CT: Yale University Press, 2009.

[115] Roper B. The History of Democracy: a Marxist Interpretation. London: Pluto Press, 2013.

[116] Rothermund D. An Economic History of India: From Pre-colonial Times to 1991. London: Routledge, 1993.

[117] Marx K. Capital: A Critique of Political Economy,1867 (transl. E Paul and C Paul). London: EP Dutton and Co, 1930.

[118] Wood EM. The Origin of Capitalism: A Longer View. London: Verso, 2002.

[119] Popkin BM. The nutrition transition in the developing world. Dev Policy World 2003;21:581–97.

[120] Monteiro CA , Cannon G, Levy RB et al. Ultra-processed foods: what they are and how to identify them. Public Health Nutr 2019;22:936–41. doi: 10.1017/S1368980018003762. 30744710 PMC10260459

[121] Baker P , Friel S. Processed foods and the nutrition transition: evidence from Asia. Obes Rev 2014;15:564–77. doi: 10.1111/obr.12174. 24735161

[122] Popkin BM , Corvalan C, Grummer-Strawn LM. Dynamics of the double burden of malnutrition and the changing nutrition reality. Lancet 2020;395:65–74. doi: 10.1016/S0140-6736(19)32497-3. 31852602 PMC7179702

[123] Wells JCK , Marphatia AA, Amable G et al. The future of human malnutrition: rebalancing agency for better nutritional health. Global Health 2021;17:119. doi: 10.1186/s12992-021-00767-4. 34627303 PMC8500827

[124] Oggioni C , Lara J, Wells JC et al. Shifts in population dietary patterns and physical inactivity as determinants of global trends in the prevalence of diabetes: an ecological analysis. Nutr Metab Cardiovasc Dis 2014;24:1105–11. doi: 10.1016/j.numecd.2014.05.005. 24954422

[125] Moodie R , Stuckler D, Monteiro C et al.; Lancet NCD Action Group. Profits and pandemics: prevention of harmful effects of tobacco, alcohol, and ultra-processed food and drink industries. Lancet 2013;381:670–9. doi: 10.1016/S0140-6736(12)62089-3. 23410611

[126] Courtwright DT. Forces of Habit – drugs and the Making of the Modern World. Cambridge, MA: Harvard University Press, 2001.

[127] Black RE , Victora CG, Walker SP et al.; Maternal and Child Nutrition Study Group. Maternal and child undernutrition and overweight in low-income and middle-income countries. Lancet 2013;382:427–51. doi: 10.1016/S0140-6736(13)60937-X. 23746772

[128] NCD Risk Factor Collaboration. A century of trends in adult human height. Elife 2016;5:e13410. doi: 10.7554/eLife.13410. 27458798 PMC4961475

[129] Wells JC , Sawaya AL, Wibaek R et al. The double burden of malnutrition: aetiological pathways and consequences for health. Lancet 2020;395:75–88. doi: 10.1016/S0140-6736(19)32472-9. 31852605 PMC7613491

[130] Wolpe H. Capitalism and cheap labour-power in South Africa: from segregation to apartheid. Econ Soc 1972;1:425–56.

[131] Turner M. Introduction. In: Turner MS (ed). From Chattel Slaves to Wage Slaves: The Dynamics of Labour Bargaining in the Americas. London: James Currey, 1995, 1–30.

[132] Marcuse P. The enclave, the citadel, and the ghetto: what has changed in the post-Fordist U.S. city. Urban Affairs Review 1997;33:228–64.

[133] Paradies Y , Ben J, Denson N et al. Racism as a determinant of health: a systematic review and meta-analysis. PLoS One 2015;10:e0138511. doi: 10.1371/journal.pone.0138511. 26398658 PMC4580597

[134] Abubakar I , Gram L, Lasoye S et al. Confronting the consequences of racism, xenophobia, and discrimination on health and health-care systems. Lancet 2022;400:2137–46. doi: 10.1016/S0140-6736(22)01989-4. 36502851

[135] Aschbacher K , O’Donovan A, Wolkowitz OM et al. Good stress, bad stress and oxidative stress: insights from anticipatory cortisol reactivity. Psychoneuroendocrinology 2013;38:1698–708. doi: 10.1016/j.psyneuen.2013.02.004. 23490070 PMC4028159

[136] Flint MS , Baum A, Chambers WH et al. Induction of DNA damage, alteration of DNA repair and transcriptional activation by stress hormones. Psychoneuroendocrinology 2007;32:470–9. doi: 10.1016/j.psyneuen.2007.02.013. 17459596

[137] Mattison SM , Smith EA, Shenk MK, Cochrane EE. The evolution of inequality. Evol Anthropol 2016;25:184–99. doi: 10.1002/evan.21491. 27519458

[138] Smith A. An Inquiry Into the Nature and Causes of the Wealth of Nations. 1776. Oxford: Clarendon Press, 1976.

[139] Childe VG. The urban revolution. Town Planning Review 1950;21:3–17.

[140] Smith EA , Codding BF. Ecological variation and institutionalized inequality in hunter-gatherer societies. Proc Natl Acad Sci USA 2021;118:e2016134118. doi: 10.1073/pnas.2016134118. 33758100 PMC8020663

[141] International Labour Organization (ILO), Walk Free, and International Organization for Migration (IOM). (2022). Global estimates of modern slavery: forced labor and forced marriage. Geneva, Switzerland: ILO. https://www.ilo.org/wcmsp5/groups/public/-ed_norm/-ipec/documents/publication/wcms_854733.pdf.

[142] Kunz N , Chesney T, Trautrims A et al. Adoption and transferability of joint interventions to fight modern slavery in food supply chains. Int J Prod Econ 2023;258:108809.

[143] Giraldeau L-A , Heeb P, Kosfeild M. Investors and Exploiters in Ecology and Economics: Principles and Applications. Cambridge, MA: MIT Press, 2017.

[144] Jaeggi AV , Boose KJ, White FJ, Gurven M. Obstacles and catalysts of cooperation in humans, bonobos, and chimpanzees: behavioural reaction norms can help explain variation in sex roles, inequality, war and peace. Behaviour 2016;153:1015–51.

[145] Boone JL. Competition, conflict, and the development of social hierarchies. In: Smith EA, Winterhalder BS (eds). Evolutionary ecology and human behavior. New York: Aldine de Gruyter, 1992, 301–337.

[146] Summers K. The evolutionary ecology of despotism. Evolution and Human Behavior 2005;26:106–35.

[147] Kaplan HS , Hooper PL, Gurven M. The evolutionary and ecological roots of human social organization. Philos Trans R Soc Lond B Biol Sci 2009;364:3289–99. doi: 10.1098/rstb.2009.0115. 19805435 PMC2781874

[148] Mangel M , Clark CW. Dynamic Modelling in Behavioral Ecology. Princeton, NJ: Princeton University Press, 1988.

