# Supplementary material for: An evolutionary perspective on social inequality and health disparities: Insights from the producer–scrounger game
Source: Evol Med Public Health. 2023 Aug 16;11(1):294–308. doi: 10.1093/emph/eoad026 (PMC10482145; doi:10.1093/emph/eoad026)

Appendix

An evolutionary perspective on social inequality and health disparities: insights from the producer-scrounger game

Jonathan Wells

The Figures produced in this article are based on the equations for the producer-scrounger game presented by Barta (1)

- Group of **G** individuals forage for **T** time units on a patch containing **F** food items
- Producers search for food and find it with rate λ
- After finding the food, each producer consumes a portion **a** (finders share) and shares the remainder (**A**, = **F-a**)
- Proportions of producers (**p**), proportion of scroungers (**1-p**) can vary

Under these assumptions, the food intake for each producer is

$$W_p(p) \quad = \quad T.\ \lambda * [a + (A/((1-p).G)+1))$$

And the food intake for each scrounger is

$$W_s(p) \quad = \quad p.G.T.\lambda(A/((1-p).G)+1)$$

At evolutionary stability, $W_p = W_s$, meaning that the frequency of each party is stable, and the frequency of producers (pf) is

$$a/F + 1/G$$

1. Barta Z; Producer-scrounger models and aspects of natural resource use. In: Giraldeau L-As (ed). *Investors and exploiters in ecology and economics: principles and applications*. Cambridge, Mass: MIT Press, 2017, 65-82.

Figure S1. Association of group size with average food intake at stable-equilibrium proportions of producers and scroungers in the population, depending on the rate of food production and the magnitude of the finder's share. Figures based on the equations of Barta (2017) as above.

In scenario (a), both *lower finder's share* and *larger group size* decrease the average food supply per individual. The horizontal line assumes a minimum food intake per individual compatible with their survival and reproduction, meaning that if this level is not reached, the entire social system will crash (producing is non-viable). As group size increases, only a finders share approaching 0.5 or higher can provide an acceptable average food intake at equilibrium. Therefore, even though the food intake of producers and scroungers at equilibrium is identical, social systems that leave producers only a small finder's share become unviable for both parties as they expand in population size.

In scenario (b), the rate at which food is produced has increased, and this increases the average food supply per individual compared to scenario (a). This solves the problem of how to achieve a viable social order as the population size expands. However, the benefits of this change accrue disproportionately to scroungers. First, scroungers can now maintain the social order even if they return a smaller finder's share to producers. Second, since the costs of producing fall only on the producers, maintaining a viable social order is achieved at the cost of the health of the producers. Scenario (b) therefore portrays a social order similar to slavery, where producers maintain the food system as a cost of coercion and poor health.

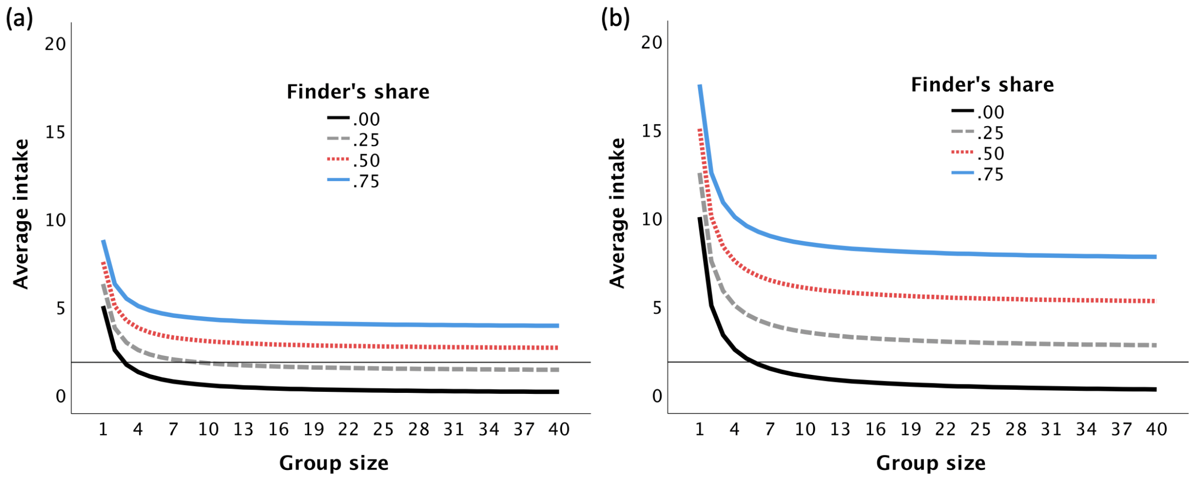

Supplement: eoad026_suppl_Supplementary_Appendix [file eoad026_suppl_supplementary_appendix.pdf]
